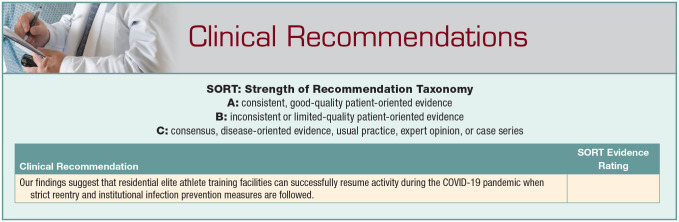# Resumption of Sport at the United States Olympic and Paralympic Training Facilities During the COVID-19 Pandemic

**DOI:** 10.1177/19417381211002761

**Published:** 2021-03-12

**Authors:** Ankit B. Shah, Dustin Nabhan, Robert Chapman, George Chiampas, Jonathan Drezner, J. Tod Olin, David Taylor, Jonathan T. Finnoff, Aaron L. Baggish

**Affiliations:** † Sports & Performance Cardiology Program, MedStar Health, Baltimore, Maryland; ‡ United States Olympic and Paralympic Committee, Colorado Springs, Colorado; § School of Public Health, Indiana University, Bloomington, Indiana; ‖ Departments of Emergency Medicine and Orthopaedic Surgery, Northwestern University, Chicago, Illinois; ¶ Department of Family Medicine, Sports Medicine Section, University of Washington, Seattle, Washington; # Department of Pediatrics, Division of Pediatric Pulmonology, Department of Medicine, Division of Pulmonary, Critical Care & Sleep Medicine, National Jewish Health, Denver, Colorado; ** Cardiovascular Performance Program, Massachusetts General Hospital, Boston, Massachusetts

**Keywords:** elite athlete training, COVID-19, myocardial injury, return-to-play

## Abstract

In this brief report, we describe the safety of reopening US Olympic and Paralympic Training facilities (USOPTFs) during the coronavirus disease 2019 (COVID-19) pandemic from July 2020 through October 2020. We evaluated the prevalence of COVID-19 infection at the time of reentry and cardiopulmonary sequelae of COVID-19 in elite athletes. All athletes returning to a USOPTF were required to go through a reentry protocol consisting of an electronic health history, a 6-day quarantine including twice-daily symptom surveys, COVID-19 polymerase chain reaction and antibody testing, physical examination, 12-lead electrocardiogram, high-sensitivity cardiac troponin I, and pulmonary function testing. Athletes with current or prior COVID-19 infection also underwent an echocardiogram, cardiology consultation, and additional testing as indicated. All athletes followed rigorous infection prevention measures and minimized contact with the outside community following reentry. At the time of this report, 301 athletes completed the reentry protocol among which 14 (4.7%) tested positive for active (positive polymerase chain reaction test, n = 3) or prior (positive antibody test, n = 11) COVID-19 infection. During the study period, this cohort accrued 14,916 days living and training at USOPTFs. Only one (0.3%) athlete was subsequently diagnosed with a new COVID-19 infection. No cardiopulmonary pathology attributable to COVID-19 was detected. Our findings suggest that residential elite athlete training facilities can successfully resume activity during the COVID-19 pandemic when strict reentry and infection prevention measures are followed. Dissemination of our reentry quarantine and screening protocols with COVID-19 mitigation measures may assist the global sports and medical community develop best practices for reopening of similar training centers.

Resumption of elite sport during the coronavirus disease 2019 (COVID-19) pandemic has generated numerous questions about optimal strategies to protect the health of competitive athletes.^9^ Return to play protocols aim to minimize the risk of COVID-19 spread among athletes during training and competition and facilitate athlete safety. Preliminary data suggest myocardial injury may occur in patients who recover from COVID-19, including mildly symptomatic and asymptomatic athletes.^7,8^ The identification of athletes at risk for adverse events attributable to cardiovascular disease is a high priority for the medical community. Accordingly, preparticipation cardiovascular screening algorithms designed for use during the COVID-19 pandemic have been published.^1,6,11^ Because of the scarcity of prospective outcomes data, these recommendations are based largely on expert opinion. Future planning for the safe resumption of competitive sport should be based on emerging scientific data. The United States Olympic and Paralympic Training facilities (USOPTFs) are the residential training base for many elite American athletes, including those preparing for the Olympic and Paralympic Games. Athletes either live on campus at the USOPTF or off-site with access to the training facility. After several months of closure, the USOPTFs reopened in July 2020. Here we report our medical protocols and early results of this experience.

## Methods

**Figure 1.**
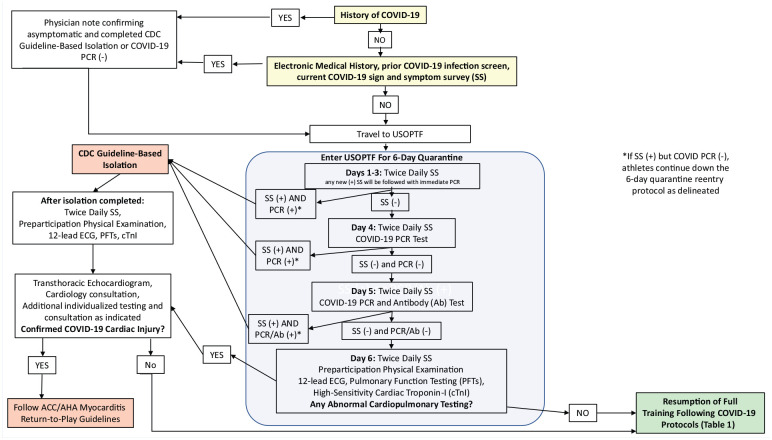
The United States Olympic and Paralympic Training facilities’ (USOPTFs) COVID-19 pandemic “return-to-training” protocol. Ab, antibody; COVID-19, coronavirus disease 2019; cTnI, cardiac specific troponin I assay; ECG, electrocardiogram; PCR, polymerase chain reaction; PFT, pulmonary function testing; SS, signs and symptom survey.

The USOPTFs’ COVID-19 pandemic “return-to-training” protocol is shown in Figure 1. Both on-site and off-site athletes completed the same protocol. Prior to arrival at an USOPTF, athletes completed an electronic health history including questions about prior COVID-19 exposure, documented COVID-19 infection, and travel in the past 14 days. This survey was designed to identify athletes with prior or current COVID-19 infection so appropriate measures can be taken prior to their travel to an USOPTF. On arrival, athletes (no other personnel) begin a 6-day quarantine during which they are required to fill out twice-daily symptom surveys. The 6-day quarantine was recommended by our Infectious Disease Advisory Group, composed of public health and infectious disease experts, after weighing the risks and benefits of various quarantine durations. During the quarantine period, athletes were provided with a personalized training program by strength and conditioning personnel that they could complete in their rooms. Equipment, such as dumbbells, stretch bands, and cardiovascular equipment (eg, bicycle trainer), were provided based on the athlete’s needs. On days 4 and 5 of the quarantine process, athletes receive a saliva-based COVID-19 polymerase chain reaction (PCR) test (Spectrum Solutions, LLC), and on day 5 they receive a COVID-19 antibody test (Premier Biotech, Inc). The tests were completed by medical personnel wearing personal protective equipment (ie, N95 mask, eye protection, gown, and gloves) in the athlete’s room. Athletes with a positive PCR are placed in isolation following Centers for Disease Control and Prevention guidelines,^2^ after which they complete the evaluation summarized below. After completing the reentry quarantine, all athletes undergo a routine elite athlete preparticipation evaluation,^4,5^ which at the USOPTF includes a resting 12-lead electrocardiogram (ECG)^10^ and pulmonary function testing.^12^ A blood assay for high-sensitivity cardiac troponin I (hs-cTnI) was also performed on all athletes postquarantine. Since data are limited on hs-cTnI levels in Olympic and Paralympic athletes, it was determined that obtaining a hs-cTnI on all of our athletes postquarantine would improve our ability to interpret test results. Athletes with a history of COVID-19 infection (positive PCR or antibody) also undergo a transthoracic echocardiogram and cardiology consultation. Additional tests (eg, cardiac magnetic resonance imaging [cMRI]) and consultations are performed as indicated. In the absence of cardiopulmonary pathology, athletes are permitted to resume training. After completing the reentry protocol, athletes are required to follow rigorous infection prevention measures (Table 1). Publication of de-identified data derived from this protocol was approved by the Mass General Brigham human subjects research committee.

**Table 1.** Key components of the United States Olympic and Paralympic Training facilities’ strategy to reduce the risk of new COVID-19 infection during residential athlete training

Twice-daily clinical sign and symptom monitoring for COVID-19
Individual living and bathroom quarters for athletes
Education on proper sneezing/coughing etiquette
Education on the need to avoid face touching
Physical distancing of at least 6 feet during rest and normal activities, and at least 12 feet during exercise
Wearing facial coverings at all times when out of living quarters except when eating or during training that would be inhibited or dangerous with a facial covering
Frequent hand washing for ≥20 seconds with soap and water when hands are visibly soiled or use of hand sanitizer with >60% alcohol content if hands are not visibly soiled
No sharing of food, water bottles, towels, or other personal hygiene products
Cleaning of equipment and facilities between athlete use with EPA approved disinfectants
Prohibition on all nonessential visitors
Limitation of on-site staff number and avoiding contact between athletes and staff
Prohibition on community activities for athletes and staff with the exception of essential off-site health care visits
Dining protocols: pickup or delivery of preordered boxed meals from the cafeteria or eating at the cafeteria in physically distanced seating locations; food in the cafeteria is served by staff behind Plexiglas barriers

COVID-19, coronavirus disease 2019; EPA, Environmental Protection Agency.

## Results

At the time of this report, 301 athletes (43% female) completed this protocol and 14 (4.7%) tested positive for active (positive PCR test, n = 3) or prior (positive antibody test, n = 11) COVID-19 infection. Two of the athletes with positive PCR tests and 2 of the athletes with positive antibody tests lived on-site, while one athlete with a positive PCR test and 9 athletes with positive antibody tests lived off-site. Each of these athletes were asymptomatic or had mild symptom burden during infection. None of these athletes had detectable hs-cTnI levels, abnormalities on ECG, echocardiography, or pulmonary function testing consistent with sequelae from COVID-19. However, 9 athletes (3.0%) without evidence of active or prior COVID-19 infection had abnormal preparticipation evaluations including 6 with abnormal resting ECGs (2.0%) and 3 with detectable hs-cTnI levels. All 3 athletes with detectable hs-cTnI levels had normal ECGs and echocardiograms, but all had exercised within 48 hours of sample collection. Repeat testing following 48 hours of no exercise yielded undetectable levels in each case. One athlete was identified as having a patent ductus arteriosus on echocardiogram. One athlete had abnormal pulmonary function testing due to known asthma. To date, this cohort has accrued 14,916 days living and training at USOPTFs. This number accounted for the time athletes were away from the USOPTFs for various reasons such as competitions. Only one (0.3%) athlete has been diagnosed with COVID-19 after successful completion of the medical reentry protocol. This athlete lived off-site. The athlete was identified after developing mild sinus congestion, prompting immediate quarantine and a COVID-19 PCR test. Following the positive PCR test, the athlete was isolated per Centers for Disease Control and Prevention guidelines, cleared repeat cardiac testing, and has returned to full training. Contact tracing was performed, and the source of the infection could not be identified. The athlete lived off-site, and while they had committed to the infection prevention measures previously outlined, it is possible the athlete unknowingly experienced a community exposure. Fortunately, none of the athletes with active or prior COVID-19 infection reported persistent or recurrent symptoms, limitations, or adverse events following resumption of training.

## Discussion

This report documents the USOPTFs’ initial experience with resumption of resident athlete training during the COVID-19 pandemic. Key findings are summarized as follows. First, the observed COVID-19 prevalence in our cohort of elite athletes returning to organized training was lower than those reported in the public media among US collegiate and professional team sport athletes.^3^ This may be explained by the fact that the majority of our cohort are athletes participating in individual sporting disciplines, who were able to train in isolation and/or with effective social distancing prior to USOPTF arrival. Second, while our sample size was small, the mild severity COVID-19 infection among young, previously healthy athletes in this cohort did not result in clinically relevant cardiovascular disease. Third, our COVID-19 risk mitigation strategy effectively prevented new COVID-19 infection among athletes. Finally, we identified no adverse events during the observed training period among athletes with prior COVID-19 infection.

Internationally, many resident sport training facilities similar to the USOPTFs will resume activity as competitions, including the 2021 Olympic Games, move forward. Dissemination of our entry screening protocols, quarantine measures, training protocol modifications, and risk mitigation strategies that have successfully prevented on-site COVID-19 outbreaks may assist the global sports and medical community with their planning. However, we acknowledge that our data are subject to several limitations. In accordance with published practice guidelines,^6^ we did not perform cMRI on our athletes with active or prior infection as all presented asymptomatic or with mild symptom burden. We may therefore have failed to detect subtle cardiac changes attributable to prior COVID-19 infection. Prospective acquisition of cMRI data coupled with a control group and clinical follow-up will be needed to determine the optimal use of this imaging modality during the COVID-19 pandemic. Additionally, our follow-up of 14,916 athlete days may not have been sufficient to capture possible adverse events. Ongoing surveillance with a commitment to disseminate additional findings is warranted. Following reentry, only athletes who developed signs and symptoms of COVID-19 were retested for infection. Although new asymptomatic cases may have been missed, an undetected outbreak in our closely monitored population is unlikely. Furthermore, ongoing COVID-19 screening adds significant cost with uncertain benefit to the COVID-19 mitigation program, making this measure unattainable for many organizations providing closed residential training. Studies replicating our results in other training settings are needed. Finally, while many of the infection prevention measures implemented as part of this protocol are broadly applicable to most settings, some of the measures taken (eg, quarantine and testing procedures) may be challenging for those with less resources such as recreational leagues and high schools.

## Conclusion

Data characterizing the USOPTFs’ early experience suggest that residential elite athlete training facilities in a controlled environment can successfully resume activity during the COVID-19 pandemic. The continued health and wellness of athletes will require a multitiered strategy that includes effective preparticipation screening, isolation of infected athletes, modified training protocols, and limited community exposure.

**Figure:**